# The Inhibitory Effect of Polyphenon 60 from Green Tea on Melanin and Tyrosinase in Zebrafish and A375 Human Melanoma Cells

**DOI:** 10.1155/2022/7739023

**Published:** 2022-09-02

**Authors:** Mehar Ali Kazi, Reshma Sahito, Qamar Abbas, Sana Ullah, Abdul Majid, Abdul Rehman Phull, Md. Mominur Rahman, Song Ja Kim

**Affiliations:** ^1^Institute of Biochemistry, University of Sindh, Jamshoro 76080, Pakistan; ^2^Department of Zoology, University of Sindh, Jamshoro 76080, Pakistan; ^3^Department of Biology, College of Science, University of Bahrain, Sakhir 32038, Bahrain; ^4^Department of Agro-Environmental Sciences, Kyushu University, Fukuoka, Japan; ^5^Department of Biochemistry, Shah Abdul Latif University, Khairpur, Pakistan; ^6^Department of Food Science and Biotechnology, Gachon University, Gyeonggi-do 13120, Republic of Korea; ^7^Department of Biology, Kongju National University, Gongju, Chungnam 32588, Republic of Korea; ^8^Department of Pharmacy, Faculty of Allied Health Sciences, Daffodil International University, Dhaka 1207, Bangladesh

## Abstract

Polyphenon 60 (PP60) from green tea has long been used as an antioxidant, anticancer, antimicrobial, and antimutagenic. *Aim of the Study*. To investigate tyrosinase inhibition-related kinetic mechanism and antimelanogenesis potential of PP60. *Materials and Methods*. The effect of PP60 on melanin and tyrosinase was evaluated in A375 melanoma cells and zebrafish embryos. The melanoma cells were treated with 20, 40, and 60 *µ*g/mL of PP60, and tyrosinase expression was induced by using L-DOPA. The western blot method was used for the evaluation of tyrosinase expression. Cell lysates were prepared from treated and untreated cells for cellular tyrosinase and melanin quantification. Furthermore, zebrafish embryos were treated with 20, 40, and 60 *µ*g/mL of PP60 and reference drug kojic acid for determination of depigmentation and melanin quantification. *In vitro* assays were also performed to examine the impact of PP60 on mushroom tyrosinase activity. To determine cytotoxicity, MTT was used against melanoma cell line A375. *Results*. PP60 showed good tyrosinase inhibitory activity with an IC_50_ value of 0.697 ± 0.021 *µ*g/mL as compared to kojic acid a reference drug with an IC_50_ value of 2.486 ± 0.085 *µ*g/mL. Kinetic analysis revealed its mixed type of inhibition against mushroom tyrosinase. In addition, western blot analysis showed that at 60 *µ*g/mL dose of PP60 significantly reduced L-DOPA-induced tyrosinase expression in melanoma cells. PP60 significantly inhibits the cellular tyrosinase (*p* < 0.05) and reduces the melanin (*p* < 0.05) contents of melanoma cells. Furthermore, PP60 was found to be very potent in significantly reducing the zebrafish embryos' pigmentation (*p* < 0.05) and melanin (*p* < 0.05) content at the dose of 60 *µ*g/mL. *Conclusions*. Our results demonstrate that PP60 has a strong potency to reduce pigmentation. It may be useful for the cosmetic industries to develop skin whitening agents with minimal toxic effects.

## 1. Introduction

In addition to protecting the skin from radiation, melanin is also accountable for the color of the skin, eyes, and hair [1]. In Human skin melasma, senile lentigines, freckles, birthmarks, ephelides, nevus, and pigmented acne scars are developed due to the accumulation of access amount of melanin [2]. In melanocytes, the tyrosinase enzyme regulates the creation of melanin. Melanocytes reside in the epidermis (basal layer) [3].

Tyrosinase inhibitors are important in the fight against excessive melanin synthesis since it is the principal enzyme involved in speeding up the process of melanin synthesis [4]. On the other hand, because free radicals may also promote melanin synthesis, different natural and synthetic antioxidant systems can scavenge such radicals that might regulate excessive melanin formation [5, 6].

Membrane-bound copper-containing glycoprotein, tyrosinase governs the two reactions, which are most essential in the formation of melanin, the monophenol to o-diphenols of ortho hydroxylation and the o-quinones corresponding oxidation. The Melanin (eumelanin and pheomelanin) production pathway in melanosomes has two steps. The first step of melanogenesis starts with tyrosine oxidation to dopaquinone catalyzed by tyrosinase, this first step is rate-limiting, and all other reactions proceed spontaneously at optimum pH. After dopaquinone formation by tyrosinase, the compound is converted to dopa and dopachrome through auto-oxidation [7].

The enzyme tyrosinase is found in fungi, plants, and mammals in abundance [8]. Melanin is a pigment that affects the color of the skin based on its type, quantity, and distribution in keratinocytes. Melanogenesis is initiated by the hydrolysis of L-tyrosine to L-dihydroxyphenylalanine (L-DOPA) followed by oxidation to DOPA quinone [9]. These reactions are catalyzed by tyrosinase. Subsequently, after a series of oxidation-reduction reactions, melanin is synthesized [10]. Tyrosinase thus plays a significant role in melanogenesis and it is a very likely target of the skin pigmentation studies performed globally [10].

Literature has reported tyrosinase as the most significant and crucial contributor to the deterioration and short half-life of fruits and vegetables during the postharvest period [11]. As a result, tyrosinase inhibitors have been of great interest to several researchers.

Natural products of herbal origin have been gaining interest among scientists in recent years for their disease prevention and health promotive roles [12–15]. Polyphenols are a class of bioactive chemicals found in fruits, vegetables, and tea [16, 17]. Tea contains a wide range of substances, particularly polyphenols, and several studies have demonstrated that these substances lower the risk of a number of illnesses. In terms of natural polyphenols, green tea extract is the most abundant source [18, 19]. Polyphenols in green tea have been extensively studied for their potential benefits, such as antibacterial, antimutagenic, and anticancer properties [20]. To the best of our knowledge, the inhibitory action of polyphenon 60 (PP60) from green tea on melanin synthesis has not been described hitherto. In the present work, PP60 in green tea has been shown to suppress melanogenesis both in the A375 melanoma cells and zebrafish embryos.

## 2. Material and Methods

### 2.1. Antityrosinase Activity Assay

The antityrosinase activity against mushroom tyrosinase (Sigma Chemical, USA) was conducted as described before [21]. Each microplate a well-contained reaction mixture comprised of tyrosinase (30 U/mL), phosphate buffer (20 mM, pH 6.8), and PP60 sample. The test plate was incubated at 25°C for 10 min. After preincubation, 20 *µ*L of L-DOPA (0.85 mM, Sigma Chemicals, USA) was poured into every well and incubated further for 20 min at similar conditions. With the help of an optimal tunable microplate reader (Sunnyvale, CA, USA), the dopachrome absorbance was measured at 475 nm. The phosphate buffer and kojic acid were used as a negative and positive control, respectively. The potential of PP60 inhibition was stated as the percentage of inhibition in activity. Whereas IC_50_ was calculated as the amount of PP60 required to produce 50% inhibition in enzyme activity. All the concentrations were tested thrice. With the GraphPad prism, the IC_50_ values were calculated. The following equation was used to compute the tyrosinase inhibition %:

(1) $$\begin{array}{c} \text{Tyrosinase inhibition}\left(\%\right)=\left[\frac{B-S}{B}\right]\times 100. \end{array}$$

In the above, the *B* is for the blank, and *S* is for the absorbance of sample.

### 2.2. Kinetics of Tyrosinase Inhibition

PP60 activity for tyrosinase inhibition was studied in a series of tests. PP60 concentrations were 0, 25, 5, 1, and 2 *µ*g/mL. L-DOPA concentrations ranged from 0.0625 to 2 mM. The method was similar to the mushroom tyrosinase inhibition test methodology. The starting linear component of optical density was evaluated up to five minutes following enzyme addition at 30 s intervals. Lineweaver‒Burk plots were used to determining the enzyme's inhibition type. To estimate the EI dissociation constant, 1/V was determined, whereas to determine the ESI dissociation constant, intercept against inhibitor concentrations was used.

### 2.3. Human A375 Melanoma Cell Culture

The cells (A375 melanoma cell) were provided by the Korean Cell Line Bank (KCLB) and cultured in DMEM with 10% heat-inactivated FBS (fetal bovine serum), 50 *µ*g/mL (streptomycin), and 50 *µ*/ml (penicillin). Incubation in a humidified CO_2_ incubator (95% O_2_, 5% CO_2_) at 37°C was done with cells seeded at a concentration of 2 × 10^5^ per ml in cell culture dishes (35 mm) for Western blotting and 96-well plates for MTT cell viability assays. After two days, the medium was refreshed, and the same protocol was followed for cell culturing until 70–75% confluence was achieved. Similarly, in a new medium, cells were allowed to develop to 70–75% confluence.

### 2.4. Cell Viability Assay

MTT test was used for cell viability assessment. The A375 cells were seeded in 96-well plates and treated to varying doses (0–70 *µ*g/mL) of PP60 for 24 hours at 37°C under CO_2_. Afterward, ten microliters of MTT reagent (0.5 *µ*g/mL) were added to each well for four hours to create purple formazan crystals. Once the formazan crystals were formed, 100 *µ*L of MTT reagent 2 (Solubilization buffer; 10% SDS with 0.01 N HCl and DMSO solution) was added, followed by overnight incubation in a CO_2_ incubator. At last, optical density at 595 nm was measured with an OPTIMax microplate reader (Sunnyvale, CA, USA). The experiment was performed three times.

### 2.5. Western Blotting

A375-melanoma cells were mixed for 24 hours with 20, 40, or 60 *µ*g/mL PP60 with or without 50 mM L-DOPA to induce tyrosinase expression. The cells were rinsed with cold 1X PBS twice, harvested and protein was extracted with radioimmunoprecipitation assay buffer (50 mM tris HCl, 150 mM NaCl, pH 7.4, 0.1% SDS, and 1% Nonidet P-40) along with protease and phosphatase inhibitors. An 8% SDS-polyacrylamide gel was used to separate equal amounts of proteins. The size-fragmented proteins were carefully transferred to nitrocellulose membranes and labeled for 3 minutes. They were then rinsed with 1X Tris-buffered saline/tween-20 (TBST-20) for 5 minutes and blocked for 1 hour in blocking buffer and 5% nonfat dried skim milk in TBST-20. After three 1 X TBST washes (30 min), upon incubation with primary antibodies for 24 hours at 4°C, membranes were rinsed and incubated for 3 hours with a 1 : 2000 dilution of horseradish peroxidase-conjugated secondary antibody followed by a second experiment the next day. In addition, the membranes were then rinsed thrice with 1X TBST and developed using a chemiluminescence kit (DOGEN, Seoul, Korea). The ImageJ program measured resolved bands for Windows (version 1.46r; NIH, USA). The protein GAPDH was used as a load check.

### 2.6. Cellular Tyrosinase Activity Assay

Cellular tyrosinase activity assay was performed by adopting the repeating approach [22]. The A375 cells were maintained and grown at the concentration of 1 × 10^4^ cells in 35 mm culture dishes. Then, cells were exposed to PP60 (20, 40, and 60 *µ*g/mL) and L-DOPA (50 mM) for 72 hours. The cells were washed twice with PBS and lysed using radioimmunoprecipitation assay buffer. The collected supernatants were incubated afterward at 37°C with 5 *µ*L of L-DOPA substrate solution for 1 hour. The tyrosinase activity of PP60 was measured through a well-plate reader (OPTIMax Tunable, Sunnyvale, CA, USA).

### 2.7. Assay of Melanin Contents on Melanoma Cells

The repeated approach of Lee et al., [22] was used to assess melanoma melanin content. The A375 cells were grown at the concentration of 1 × 10^4^ cells in 35 mm cell culture dishes. The impact of PP60 on melanin content was studied in cells by exposing to 0 to 60 *µ*g/mL of PP60 for 72 hours. After the predetermined treatment of PP60, the cells were harvested and collected in PCR tubes at the speed of 1000 rpm for 10 minutes. Afterward, the pellet was dissolved in a solution of 1 N NaOH for 90 minutes at 60°C. A microplate reader measured the absorbance of the supernatant at 450 nm (OPTIMax Tunable, Sunnyvale, CA, USA).

### 2.8. The In Vivo Zebrafish Assay of Depigmentation

The *in vivo* depigmentation approach in zebrafish was conducted through a previously reported slightly modified method [23].

#### 2.8.1. Zebrafish Husbandry

The animals were obtained from a commercial vendor and acclimatized for a month at 28 ± 2°C with a photoperiod of 14 h light and 10 h dark. Fresh brine shrimp larvae and dry food were provided twice a day. The fish were kept alive through chemico-biological, mechanical filtration, and aeration. Induced spawning was produced in the presence of light in the morning. All procedures were performed according to the principles of laboratory animal care (NIH publication 85–23, revised 1985), and Kongju National University's Institutional Review Board approved the study (IRB number 2011-2). The collection of embryos took 30 minutes.

#### 2.8.2. PP60 Treatment and Phenotype-Based Evaluation

In 200 *µ*L of E3 medium (Sodium chloride 5 mM, KCl 0.17 mM, CaCl_2_ 0.33 mM, Magnesium sulfate 0.33 mM), 2-3 synchronized embryos were pipetted per well into 96-well plates. After fertilization, they were exposed to the E3 medium for 9 to 72 hours, totaling 63 hours. Kojic acid was used as a positive control. With the stereomicroscope (SMZ745T, Nikon, Japan), anesthesia was administered to embryos with dechorionated cell bodies using tricaine methanesulfonate (150 mg/L) solution (Sigma, Chemicals, USA). ImageJ software was used to measure pixels (National Health Institution of USA).

### 2.9. Identification of Melanin Contents from Zebrafish

The approach of and Baek et al. [1, 24] over in vivo depigmentation of zebrafish embryos was used in this study. The total synchronized 20 embryos were mixed with 20, 40, and 60 *µ*g/mL of PP60, and the reference medication was 3 mL of kojic acid in E3 medium. In tricaine MS-222 solution, the embryos were anesthetized at 72 hours post fertilization. After anesthesia, the embryos were washed thrice in an E3 medium. Additionally, both untreated and treated embryo eyes were removed. A homogenized embryo extract (pellet) was then prepared via centrifugation and homogenization. Analyzing the absorbance at 405 nm in comparison to a synthetic melanin standard curve allowed us to determine the melanin content. All experiments were performed in triplicate.

### 2.10. Statistical Analysis

With the Statistical Package for Social Sciences (SPSS version 16.0 Inc. Chicago, Illinois, USA), data were analyzed by one-way analysis of variance (ANOVA). A posthoc Tukey‒Kramer test was used when the normality test failed the Ranks test. The value difference, *p* < 0.05 was considered statistically significant.

## 3. Results and Discussion

Skin pigmentation is an ever-vibrant field of research around the globe and the multimillion-dollar cosmetic industry has undergone a tremendous surge in its research and development sector. This has led to a staggering rise in the variety of skin products available in the market [9, 25]. There has been a great interest in the use of plant extracts and compounds isolated from natural sources including secondary metabolites, i.e., polyphenols to investigate new bioactive compounds targeting melanin production [26]. Tyrosinase enzyme along with TRP-1 and 2 proteins are the key players in the melanogenesis pathway and these can be easy targets for drug candidates affecting pigmentation. The suppression of this enzyme and the proteins is a highly effective way to decrease melanin synthesis [27].

### 3.1. Antityrosinase Activity Assay

The antityrosinase inhibitory potential of PP60 was evaluated using an *in vitro* assay. For the determination IC_50_ value, different doses of PP60 ranging from 0 to 10 *µ*g/mL were used in the experiment. According to the results, PP60 possessed a very competitive inhibition counter to that of mushroom tyrosinase exhibiting an IC_50_ value of 0.697 ± 0.021 *µ*g/mL compared to kojic acid with an IC_50_ value of 2.486 ± 0.085 *µ*g/mL. The larger quantity of catechin in PP60 reported by Jung et al. [28] showed the therapeutic benefit of PP60 on acne, it may be due to the participation of catechins in tyrosinase inhibition. Tyrosinase catalyzes the conversion of tyrosine to L-DOPA which finally converts to DOPA quinone. Thus, most skin-lightening treatments block tyrosinase to reduce melanogenesis. The antioxidative ability of tea polyphenols has been partially credited with the potential health advantages linked with tea drinking. Green tea has recently been linked to improved overall antioxidative status and protection against oxidative damage in humans when ingested as part of a balanced, regulated diet [1].

### 3.2. Kinetic Study

**Figure 1:**
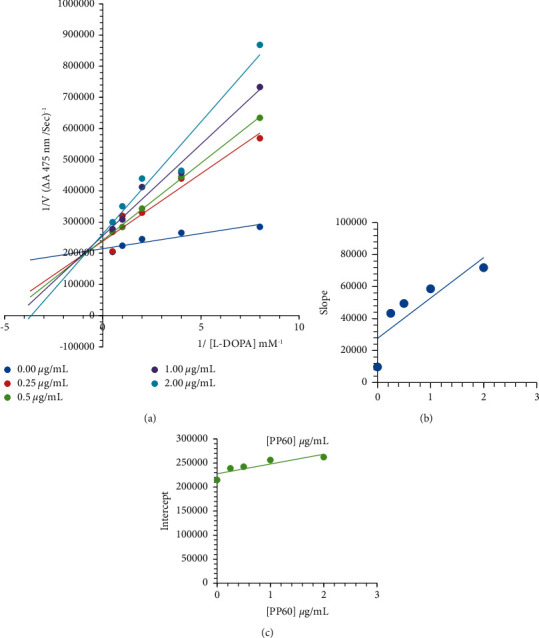
A plot of the Lineweaver‒Burk plot for tyrosinase inhibition of PP60. (a) Accordingly, the concentrations of PP60 were 0, 0.25, 0.5, 1.0, and 2.0 *µ*g/mL. The L-DOPA concentrations of the subjects were, respectively, 0.0625, 0.125, 0.25, 0.5, 1, and 2 mM. The insets (b) are plots of slopes and (c) of vertical intercepts versus various doses of PP60 to evaluate inhibition constants. Using the least square fit with linear least squares, the lines were drawn.

The manner of PP60 inhibition against mushroom tyrosinase was studied kinetically (Table 1). By measuring EI and ESI constants, PP60 was tested for its ability to inhibit the free enzyme and the enzyme-substrate complex.  Figure 1 shows the Lineweaver–Burk plot of 1/V versus 1/[L-DOPA] at various PP60 concentrations, which exhibits a succession of straight lines (*A*). PP60 intersected the second quadrant in Figure 1(a). In the rising PP60 concentrations, the decrease of Vmax occurred with the increase of Km. PP60 inhibits tyrosinase in two ways: by competitively creating enzyme inhibitor complexes and noncompetitively interrupting enzyme-substrate inhibitor complexes. Secondary slope vs. PP60 concentration plots indicated EI dissociation constants Ki Figure 1(b), whereas secondary intercept versus PP60 concentration plots gave ESI dissociation constants Ki′ Figures 1(b) and 1(c). The smaller the Ki than Ki′ indicated better enzyme-PP60 binding and so favored competitive over noncompetitive mechanisms (Table 1).

### 3.3. Cellular Viability Results of PP60

**Figure 2:**
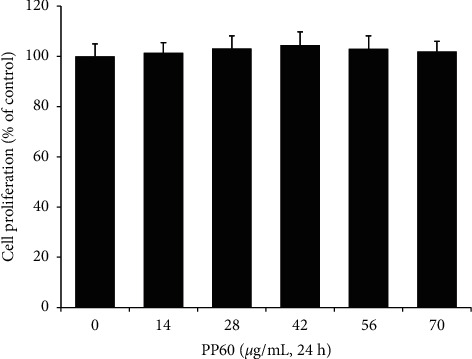
The cell viability of PP60 was evaluated by treating A375 melanoma cells for 24 hours with various concentrations and examining cytotoxicity using the viability assay kit. All of the results and values are represented as the average of triplicate experiments with standard deviation.

The cellular viability (MTT assay) was performed for the detection of the cellular toxicity of PP60 targeting A375 cells. The A375 melanoma cells were evaluated for 24 h with different concentrations (0–70 *µ*g/mL). The results confirmed the noncytotoxicity of PP60 compared to those of control (Figure 2). However, an insignificant decrease in cell viability was demonstrated by PP60 in a concentration dependent manner as shown in Figure 2. The cells with no treatment of PP60 were supposed to be100% viable.

### 3.4. PP60 Decreases the Expression of the Enzyme Tyrosinase

**Figure 3:**
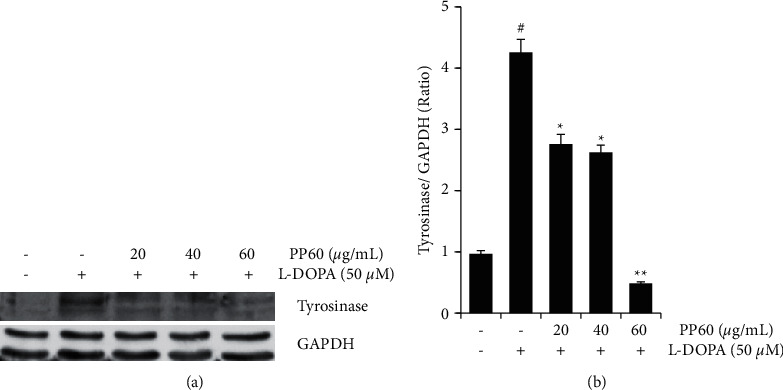
The protein expression of the enzyme tyrosinase was analyzed in comparison with GAPDH on the A375 melanoma cell line. The cells (a, b) were targeted to different L-DOPA concentrations with and without (20, 40, and 60 g/mL) of PP60 for 24 hours. From the western blot analysis, the appearance of tyrosinase was clarified with the help of GAPDH as a loading control. The differences were considered significant at the level of # *p* < 0.001 for L-DOPA induced in comparison to normal control and ^*∗*^*p* < 0.05, ^*∗∗*^*p* < 0.001 PP60 inhibited tyrosinase expressions in comparison to L-DOPA induced.

In western blots, tyrosinase enzyme expression was assessed. PP60 was applied to A375 melanoma cells at 0, 20, 40, and 60 *µ*g/mL to examine how it affects tyrosinase activity. Tyrosinase expression was induced with L-DOPA. Results showed the significant induction of tyrosinase expression (4.39 fold, *p* < 0.001) in L-DOPA treated cells (Figure 3) compared to normal control. Most significant (*p* < 0.001) inhibition of tyrosinase expression occurred at the dose of 60 *µ*g/mL in comparison to L-DOPA enhanced expression, while moderate significant (*p* < 0.05) reduction of tyrosinase expressions were found at the 20 and 40 *µ*g/mL (Figures 3(a) and 3(b)). Inhibiting melanin formation has two basic modes of action. First, it suppresses the tyrosinase enzyme activity *in vitro*, and then it reduces tyrosinase protein levels in cells. Hydroxyquinone, arbutin, and kojic acid are examples of the first technique [29]. However, many medications work by blocking tyrosinase expression in cells [30]. However, our data indicated that PP60 inhibits both expressions of tyrosinase in L-DOPA-induced cells and *in vitro* enzyme activity against mushroom tyrosinase.

### 3.5. Results of PP60 Effect on Cellular Tyrosinase from A375 Melanoma Cells

**Figure 4:**
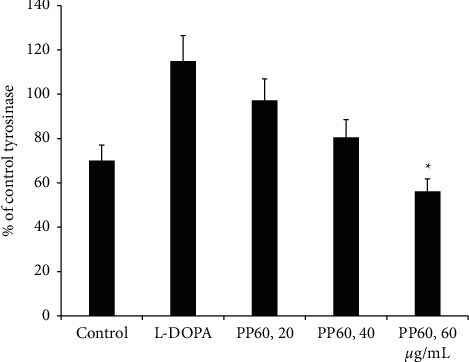
The PP60 was evaluated against the cellular tyrosinase. The cells of A375 of melanoma were evaluated with ranges of concentration from 20, 40, and 60 *µ*g/mL of PP60 along with 50 mM L-DOPA for tyrosinase induction. ^*∗*^*p* < 0.05; values expressed as a % control.

The PP60 effect on the activity of cellular tyrosinase was analyzed; the lysates of the cells were prepared from the A375 melanoma cells, mixed for 72 h with 20, 40, and 60 *µ*g/mL of PP60 and 50 *µ*M of L-DOPA. The results showed the significant downregulation of cellular tyrosinase at the concentration of 60 *µ*g/mL PP60 compared to control and L-DOPA exposed cells. The above results together with these results (Figure 4.) consistent with that PP60 have both modes of inhibition of tyrosinase indirect *in vitro* and in cells.

### 3.6. Melanin Content from Melanoma Cells

**Figure 5:**
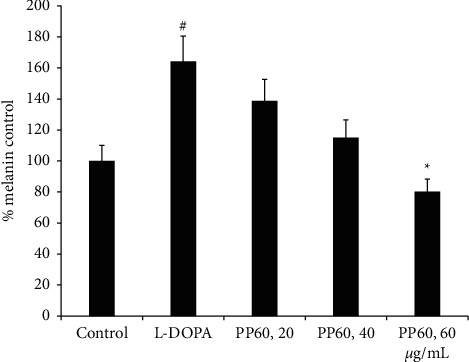
PP60 effect against melanin assay was studied. PP60 was added in varying concentrations to A375 melanoma cells, 20, 40, and 60 *µ*g/mL. Percentage values are shown as % control. ^#^ Showed significantly higher melanin compared to control (*p* < 0.01), while ^*∗*^ representing PP60 mediated reduction in melanin compared to L-DOPA group (*p* < 0.05).

In the Mammalian skins, melanin contributes a pivotal role in color determination. The effects of PP60 as well as L-DOPA and varying concentration ranges of PP60, on melanin, were examined in the melanoma cells. For the 3 days of treatment with 50 µM of L-DOPA, a significant (*p* < 0.05, Figure 5) increase was determined. PP60 decreased the melanin contents significantly as the concentration of PP60 increased; also, the prominent decrease was investigated at 60 *µ*g/mL compared to L-DOPA treated with the control cells. As Figure 5 indicates that melanin content from melanoma cells matched with the tyrosinase inhibitory activities of 60 *µ*g/mL of PP60, it might be melanin downregulation due to the inhibition of tyrosinase. The melanin activity of the 95% tea ethanolic extracts was higher in vitro. This occurrence might be explained by the extract's increased concentration of antioxidant chemicals, including natural plant polyphenols. [31].

### 3.7. PP60 Reduces the Melanogenesis in Zebrafish Embryos

**Figure 6:**
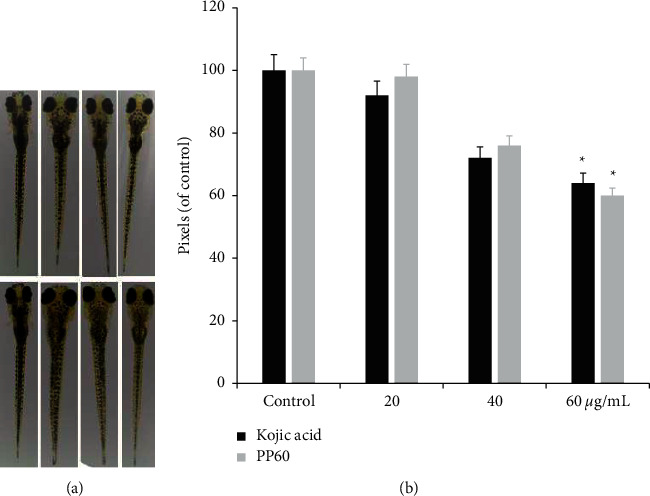
The depigmentation effect of PP60 on zebrafish. Positive control, kojic acid, and embryos treated with sample PP60 at 20, 40, and 60 *µ*g/mL. (a) Representative picture of the pigmentation levels of zebrafish treated with PP60 and kojic acid. (b) Pixel comparison of PP60's and koji acid's depigmenting effect at the level of ^*∗*^*p* < 0.05.

It is very important to study zebrafish as a vertebrate model for its similar gene sequence to humans [32]. Because of these similar and beneficial gene sequences, through *in vivo* assays of zebrafish embryos, we determined the potency of PP60's depigmentation ability. The inhibition effects of PP60 on zebrafish pigmentation were studied using PP60 at different concentrations (20, 40, and 60 *µ*g/mL) as well as kojic acid at the same concentration as the positive control.  Figure 6(a) shows a significant decrease in pigment level among zebrafish (*p* < 0.05) while Figure 6(b) shows a reduction of 36% in pigmentation with 60 *µ*g/mL kojic acid (positive control). Phenolic compounds are well-known for their wide array of biological functions [33, 34] and antioxidant polyphenols in green tea are well-known for quenching free radicals [35]. Reactive oxygen species (ROS) scavenging and interfacing with melanogenic regulators have been shown to have antimelanoma properties in the literature [22].

### 3.8. Effect of PP60 on Zebrafish Melanin Contents

**Figure 7:**
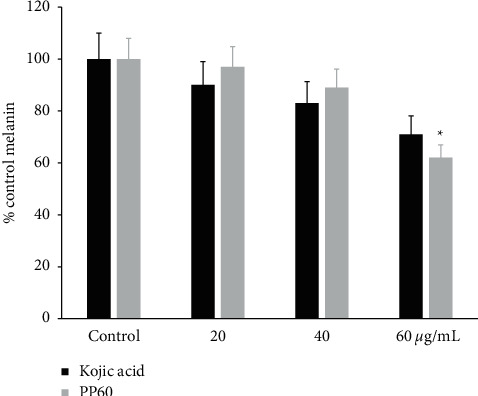
The PP60 and its effect on melanin contents were measured using embryos of the zebrafish. The positive control kojic acid together with the embryos of the zebrafish was evaluated with 20, 40, and 60 *µ*g/mL of PP60. Percentages of control are presented. ^*∗*^*p* < 0.05.

The content of melanin was determined from zebrafish embryos. There is a significant reduction of melanin occurs (*p* < 0.05) when 60 *µ*g/mL of PP60 is added to the Zebrafish embryos compared to control kojic acid (a reference drug). Melanin contents moderately decreased in the kojic acid-treated embryos, while PP60 decreased the melanin more than the kojic acid (Figure 7). As early as 28 hpf, the first zebrafish larval melanocytes begin to develop, and by 60 hpf, 460 postmitotic melanocytes are contributing to the formation of the pigment pattern [36]. There is a strong correlation between the decrease in melanin concentration and the loss of dendritic morphology [37].

## 4. Conclusions

The current study demonstrated that PP60 possesses a remarkable capacity to inhibit tyrosinase activity both competitively and noncompetitively. Moreover, PP60 also inhibited melanin synthesis in melanoma cell lines as well as in zebrafish embryos. At a concentration of 60 *µ*g/mL, PP60 caused a significant decrease in melanin synthesis in the melanoma cells as well as inhibition of tyrosinase activity. The results were further confirmed by the findings of western blot analysis. The tyrosinase inhibition and, subsequently, the pigmentation lowering activity of PP60 was assessed in vitro followed by in vivo examination. The toxicity profile of PP60 was also evaluated, and the results of cell viability revealed no cytotoxicity against A375 melanoma cells and the zebrafish. This further confirmed the selective action of PP60 and adds sustenance to the possibility of developing minimally cytotoxic antimelanogenic drugs.

## Figures and Tables

**Table 1:** The kinetic parameters of the L-DOPA activity of mushroom tyrosinase, in the presence of different concentrations of PP60.

Dose (*µ*g/mL)	*V*_max_ (ΔA/Sec)	km inhibition (mM) type	*Ki* (*µ*g/mL)	*Ki′* (*µ*g/mL)
0.00	4.891 × 10^−6^	0.043		
0.25	4.848 × 10^−6^	0.161		
0.5	3.727 × 10^−6^	0.181 mixed	1.125	11.35
1.0	3.599 × 10^−6^	0.204		
2.0	3.333 × 10^−6^	0.235		

*V*_max,_ Km, and Ki are equal to reaction velocity, Michaelis‒Menten constant, and El dissociation constant, respectively.

## Data Availability

The data used to support the findings of this study are available from the corresponding author upon request.
